# Posttranslational Modifications of the Histone 3 Tail and Their Impact on the Activity of Histone Lysine Demethylases *In Vitro*

**DOI:** 10.1371/journal.pone.0067653

**Published:** 2013-07-02

**Authors:** Brian Lohse, Charlotte Helgstrand, Jan B. L. Kristensen, Ulrike Leurs, Paul A. C. Cloos, Jesper L. Kristensen, Rasmus P. Clausen

**Affiliations:** 1 Department of Drug Design and Pharmacology, Faculty of Health and Medical Sciences, University of Copenhagen, Copenhagen, Denmark; 2 Biotech Research & Innovation Centre, University of Copenhagen, Copenhagen, Denmark; Peking University Health Science Center, China

## Abstract

Posttranslational modifications (PTMs) of the histone H3 tail such as methylation, acetylation and phosphorylation play important roles in epigenetic signaling. Here we study the effect of some of these PTMs on the demethylation rates of methylated lysine 9 *in vitro* using peptide substrates mimicking histone H3. Various combinations with other PTMs were employed to study possible cross-talk effects by comparing enzyme kinetic characteristics. We compared the kinetics of histone tail substrates for truncated histone lysine demethylases KDM4A and KDM4C containing only the catalytic core (cc) and some combinations were characterized on full length (FL) KDM4A and KDM4C. We found that the substrates combining trimethylated K4 and K9 resulted in a significant increase in the catalytic activity for FL-KDM4A. For the truncated versions of KDM4A and KDM4C a two-fold increase in the catalytic activity toward bis-trimethylated substrates could be observed. Furthermore, a significant difference in the catalytic activity between dimethylated and trimethylated substrates was found for full length demethylases in line with what has been reported previously for truncated demethylases. Histone peptide substrates phosphorylated at T11 could not be demethylated by neither truncated nor full length KDM4A and KDM4C, suggesting that phosphorylation of threonine 11 prevents demethylation of the H3K9me3 mark on the same peptide. Acetylation of K14 was also found to influence demethylation rates significantly. Thus, for truncated KDM4A, acetylation on K14 of the substrate leads to an increase in enzymatic catalytic efficiency (*k*_cat_/*K*_m_), while for truncated KDM4C it induces a decrease, primarily caused by changes in *K*_m_. This study demonstrates that demethylation activities towards trimethylated H3K9 are significantly influenced by other PTMs on the same peptide, and emphasizes the importance of studying these interactions at the peptide level to get a more detailed understanding of the dynamics of epigenetic marks.

## Introduction

The tails of the histone proteins are subject to a plethora of posttranslational modifications (PTMs), which have been linked to chromatin remodeling, transcriptional regulation, DNA replication and repair [1]–[4]. The chemical modifications include acetylation of lysine (K_(ac)_) residues, different methylation states of lysine (Kme_n_) and arginine (Rme_n_) residues, phosphorylation of serine (S_(ph)_) and threonine (T_(ph)_) residues. Alone, or in combination, these PTMs can lead to either gene activation or repression, and it has been proposed that these distinct patterns of PTMs constitute part of a histone code [2]. A large number of enzymes involved in epigenetic regulation have been reported catalyzing the formation and removal of histone marks, commonly referred to as writers and erasers. One of these is the family of JmjC histone lysine demethylases (KDMs) [3], [4]. It has been demonstrated that single PTMs of the histone tails or combinations thereof can affect the activity of these enzymes [5]–[7]. Thus, a PTM in one part of the histone tail can influence how another PTM is recognized and processed [8]. Unraveling these combinatorial effects, previously termed cross-talk, is important for the understanding of the dynamics of epigenetic regulation. Many investigations have aimed at finding interactions between histone marks in cells where hundreds or thousands of marks are analyzed simultaneously, thus investigating a general mechanism at a global level spanning the entire chromatin. Cross-talk combination effects could result in different outcomes on a molecular, cellular, phenotypic and genomic level and have different causes. Thus, cross-talk could origin from two or more PTMs on the same histone tail or due to a PTM at a neighboring histone tail. The outcome could be altered enzymatic activity or the recruitment of epigenetic proteins could be affected. Thus, *in vitro* studies that systematically look for cross-talk effects at the single histone tail level are important to unravel effects that may not be visible at a global cellular level, but could be important at the local level of individual genes. In the present study, we demonstrate how different PTMs can have significant effects on the *in vitro* demethylation activity of the JmjC histone demethylases KDM4A and KDM4C of lysine 9 in histone H3 (H3K9).

**Figure 1:**
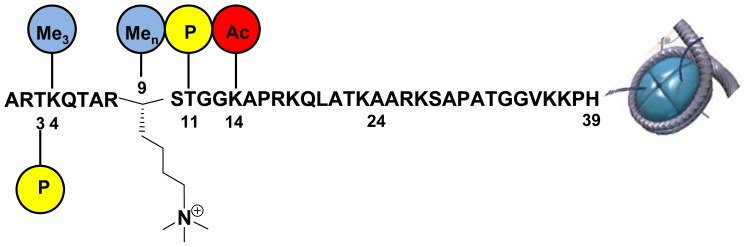
The PTMs investigated in this study for their cross-talk effects on demethylation of H3K9me3 (shown) or H3K9me2 by cc-KDM4A/cc-KDM4C and FL-KDM4A/FL-KDM4C. Methylated lysine (blue circles), phosphorylated threonine (yellow circle) and acetylated lysine (red circle) were investigated.

Four PTMs were investigated *in vitro* on a truncated histone 3 (H3) tail peptide, in order to determine if cross-talk between these PTMs affects the demethylation of tri- or dimethylated lysine 9 of histone 3 (H3K9me3/me2) (figure 1). Truncated constructs of KDM4A and KDM4C containing only the catalytic cores (cc-KDM4A/cc-KDM4C) have previously been shown to demethylate H3K9me3/me2 and H3K36me3/me2 [9], [10]. However, no enzyme kinetic analyses have been reported for full length KDM4A and KDM4C (FL-KDM4A/FL-KDM4C). All enzyme kinetic studies on the KDM-family has until now been based on the truncated proteins. In this study we found it useful to extend part of the study to include full length KDMs as well, since these enzymes feature various protein domains (in addition to the catalytic JmjC domain) known to interact with histone tail PTMs, e.g. PHD-domains [5]. The four investigated PTMs were phosphorylation of T3 and T11, trimethylation of K4 and acetylation of K14 (figure 1).

H3K9me3 is a well-known repressive mark that has been associated to heterochromatin and linked to oncogene induced cellular senescence [11]–[13]. Over-expression of KDM4 demethylases have been observed in several types of cancer and the link to tumor growth has been supported by several lines of evidence [9], [14], [15]. Thus, these enzymes are important for normal cellular functions, and have attracted growing attention as interesting therapeutic targets, in the pursuit of potential new anticancer drugs [16]. Phosphorylation of H3T3 has been shown to be important for normal metaphase chromosome alignment [17] and phosphorylation of H3T11 appears to be a key modification during meiosis [18] and has previously been reported to accelerate the demethylation reaction rate by KDM4C [6]. Trimethylated H3K4 has been reported to be present at the vast majority of euchromatic genes occupied by H3K9me3 [7], [19] and acetylation of H3K14 is critical for DNA damage checkpoint activation [20]. Thus, for various reasons these PTMs seemed interesting to investigate.

In a previous study of the *in vitro* effect of histone tail truncation, we reported that the kinetics of K9 demethylation by cc-KDM4A and cc-KDM4C were comparable until the peptide chain length was less than 8 amino acid residues [21]. All truncated histone tails employed in this study consisted of the 24 first amino acids from the N-terminus of H3, featuring either single modifications e.g. H3_(1–24)_K9me3 or double modifications e.g. H3_(1–24)_K9me3-T11_(ph)_.

## Results

**Figure 2:**
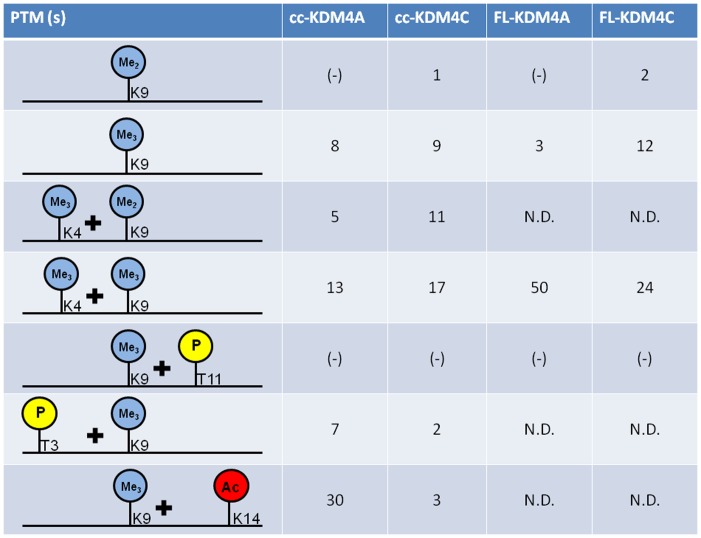
Summary of the enzymes, substrate modifications and corresponding effects. The figure shows the PTM combinations and their cross-talk effects as a function of enzymatic catalytic efficiency (*k*_cat_/*K*_m_) in (min^−1^ * µM^−1^) * 10^3^, for truncated and full length enzymes on truncated histone tails. (−): no detectable enzymatic catalytic activity. N.D: Not determined.

A summary of the modifications and their cross-talk effects are presented in figure 2.

### Demethylation of H3_(1–24)_K9me3/me2 by FL-KDM4A/KDM4C

**Figure 3:**
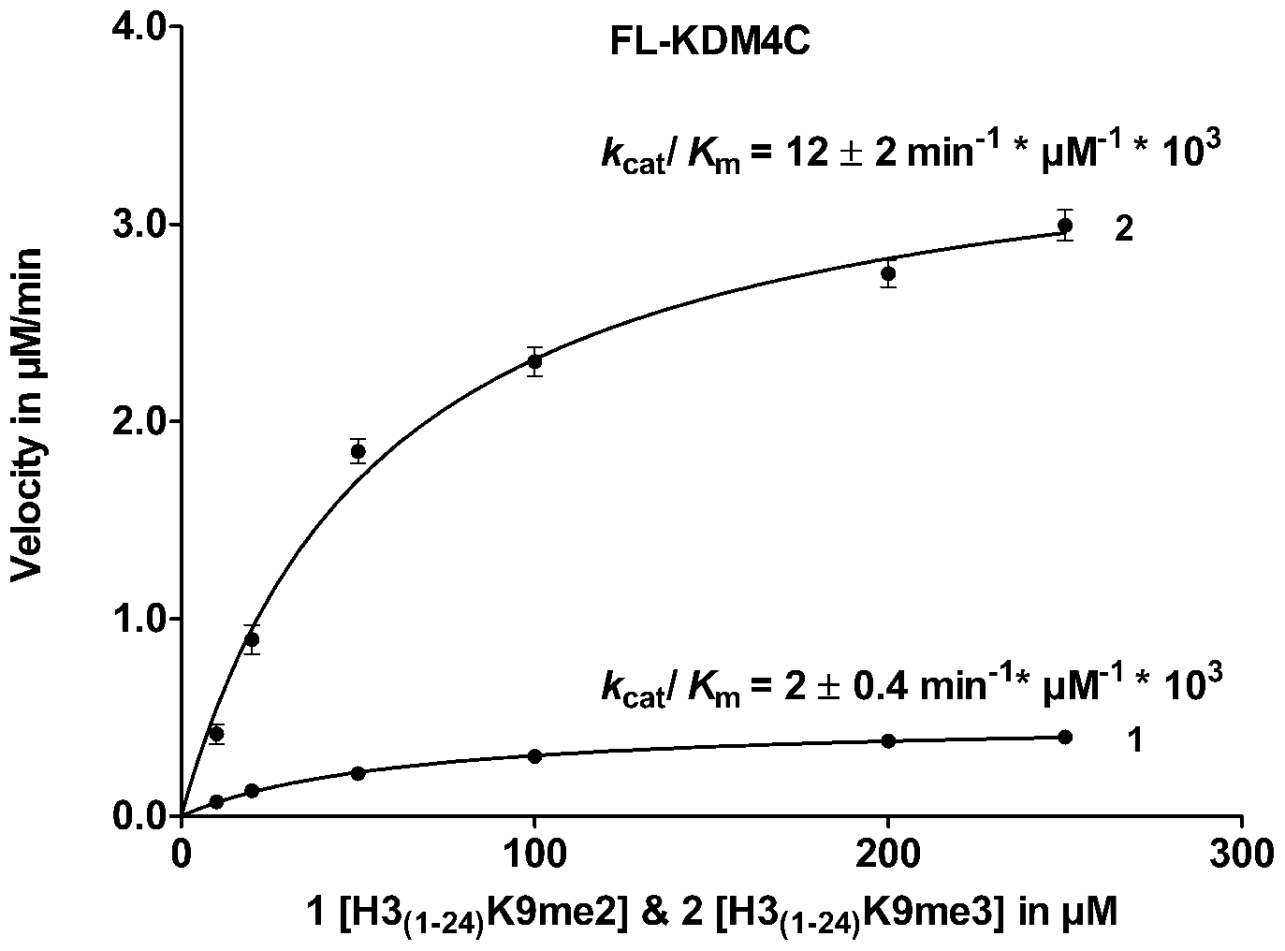
Enzyme kinetics for FL-KDM4C. H3K9me2 vs. H3K9me3 peptide substrate analogues, depicting the different *k*_cat_/*K*_M_-values of H3_(1–24)_K9me2 (**1**) and H3_(1–24)_K9me3 (**2**) for FL-KDM4C.

For both FL-KDM4A and FL-KDM4C, a significant difference in catalytic activity towards the di- and trimethylated species were observed with a preference for the H3K9me3 over the H3K9me2 (figure 3).

The difference in kinetic parameters for H3_(1–24)_K9me2 and H3_(1–24)_K9me3 at the full length enzymes were comparable to those previously reported for truncated enzymes [21], [22]. For FL-KDM4A, the formation of H3_(1–24)_K9me1 upon demethylation of H3_(1–24)_K9me2 was too low to be detected in the FDH-assay under the used conditions.

### Phosphorylation of Threonine 11 (H3T11) Inhibits Demethylation of H3K9 by KDM4A and KDM4C

Phosphorylation of threonine 11 H3_(1–24)_K9me3-T11_(ph)_, resulted in no detectable turnover of this peptide by neither truncated nor full length enzymes as assayed by both MALDI-TOF-MS (figure S1 in File S1) and FDH-assay. This result indicates that phosphorylation of threonine 11 prevents demethylation at K9me3. To investigate if H3_(1–24)_K9me3-T11_(ph)_ could bind to the enzyme and act as an inhibitor, a competition experiment with H3_(1–24)_K9me3 was carried out. It was observed that the catalytic activity towards H3_(1–24)_K9me3 was unchanged in the presence of H3_(1–24)_K9me3-T11_(ph)_, indicating that H3_(1–24)_K9me3-T11_(ph)_ does not act as an inhibitor (results not shown).

**Figure 4:**
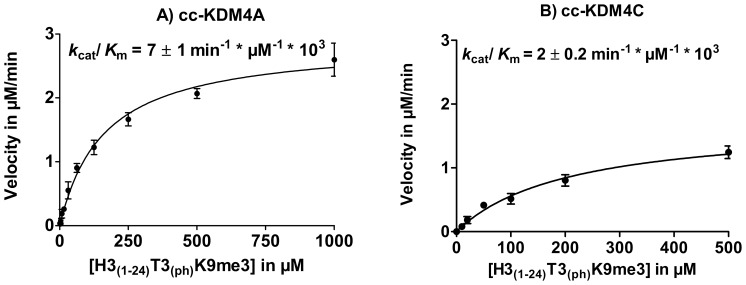
Enzyme kinetics for cc-KDM4A and cc-KDM4C using H3_(1–24)_ T3_(ph)_ -K9me3. A) Enzyme kinetics for cc-KDM4A and B) Enzyme kinetics of cc-KDM4C.

However, phosphorylation of threonine 3 in combination with lysine 9 trimethylation H3_(1–24)_T3_(ph)_-K9me3 did not prevent demethylation by cc-KDM4A and cc-KDM4C, but displayed kinetics similar to H3_(1–24)_K9me3 (figure 4) although a 5-fold decrease in catalytic activity was observed for cc-KDM4C, primarily due to a change in *K*_m_ (see table 1).

**Table 1:** Substrate selectivity of the human cc-KDM4A and cc-KDM4C histone demethylases.

Truncated Enzymes	cc-KDM4A			cc-KDM4C		
Kinetic parameters	*K*_m_	*k*_cat_	*k*_cat_ */K*_m_	*K*_m_	*k*_cat_	*k*_cat_ */K*_m_
**H3_(1–24)_K9me2**	Below detection	Below detection	Below detection	79±10	0.1±0.01	1.3±0.2
**H3_(1–24)_K9me3**	131±16	1.1±0.04	8.4±1.1	55±11	0.5±0.04	9±1
**H3_(1–24)_K4me3K9me3**	82±11	1.1±0.04	13.4±1.9	70±13	1.2±0.1	17±2
**H3_(1–24)_K9me3K14_(ac)_**	33±4	1.0±0.03	30.3±3.8	35.2±20	0.1±0.01	2.8±0.2
**H3_(1–24)_K9me3T11_(ph)_**	0	0	0	0	0	0
**H3_(1–24)_K9me3T11_(ph)_** then H3_(1–24)_K9me3*	132±23	1.4±0.08	10.6±1.9	43±22	0.3±0.05	6.3±0.5
**H3_(1–24)_T3_(ph)_K9me3**	162±15	1.2±0.04	7.4±0.7	207±50	0.38±0.04	1.8±0.1

The different kinetic parameters *K*_m_, *k*_cat_ and *k*_cat_/*K*_m_ are shown for comparison. 0 means no demethylation could be detected.

### Acetylation of Lysine 14, H3K9me3 in Combination with H3K14_(ac)_

**Figure 5:**
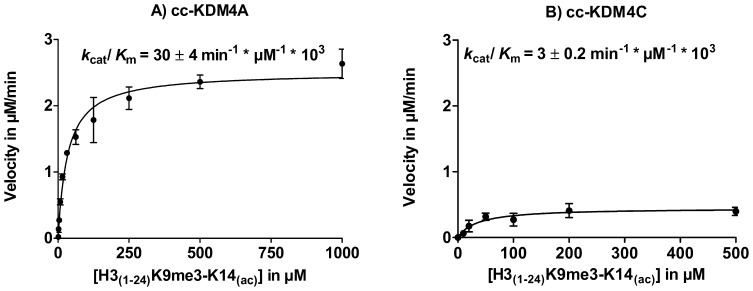
PTM cross-talk between K9me3 and K14_(ac)_ on cc-KDM4A and cc-KDM4C. Enzyme kinetics with H3_(1–24)_K9me3-K14_(ac)_ for cc-KDM4A A) and cc-KDM4C B).

Using histone peptides containing H3K9me3 in combination with acetylation of K14 (H3_(1–24)_K9me3-K14_(ac)_) as substrates resulted in a significant change of the demethylation activity of cc-KDM4A (figure 5A) and cc-KDM4C (figure 5B) compared to the control peptide H3_(1–24)_K9me3. For cc-KDM4A, an increase in the enzymatic catalytic efficiency was detected, while for cc-KDM4C a decrease was observed (see figure S2 in File S1 for MALDI-TOF-MS). Interestingly, this resulted in a 10 fold difference in a catalytic activity between cc-KDM4A and cc-KDM4C with substrate H3_(1–24)_K9me3-K14_(ac)_, see table 1.

### H3K4me3 in Combination with H3K9me3

**Figure 6:**
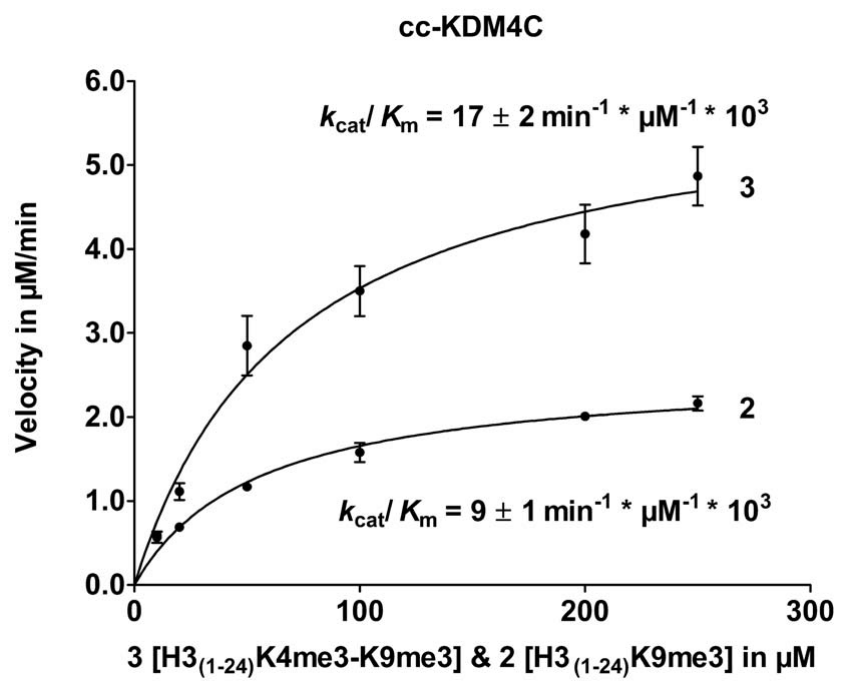
PTM cross-talk between K4me3 and K9me3 on cc-KDM4C. Enzyme kinetics for trimethylated substrate H3_(1–24)_K9me3 (**2**) and bis-tri-methylated substrate H3_(1–24)_K4me3-K9me3 (**3**).

The peptide substrate containing a trimethylated K4 in combination with H3K9me3 (H3_(1–24)_K4me3-K9me3) resulted in an increase of the enzymatic catalytic efficiency for cc-KDM4C (figure 6) (see figures S3 and S4 in File S1 for MALDI-TOF-MS). The H3_(1–24)_K4me3 peptide was also characterized for both enzymes to investigate the possible demethylation of trimethylated H3K4 (see figure S5 in File S1 for MALDI-TOF-MS).

The kinetic analyses of the substrate specificity of cc-KDM4A show a *k*_cat_/*K*_m_ of 8.4±1.1 min^−1^ * µM^−1^ * 10^3^ for substrate H3_(1–24)_K9me3, while the bis-trimethylated substrate **(**H3_(1–24)_K4me3-K9me3) gave a comparable *k*_cat_/*K*_m_ value (13±2 min^−1^ * µM^−1^ * 10^3^).

**Figure 7:**
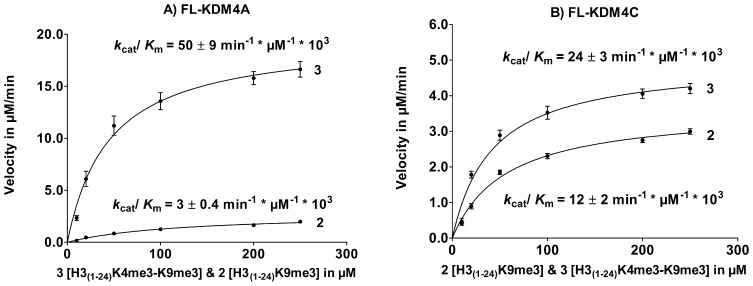
PTM cross-talk between K4me3 and K9me3 on FL-KDM4A and FL-KDM4C. Enzyme kinetics of A) FL-KDM4A and B) FL-KDM4C for H3_(1–24)_K9me3 (**2**) and H3_(1–24)_K4me3K9me3 (**3**).

It has been shown for PHF8 [5] that the presence of a PHD domain is important for trimethylated K4 substrate to be able to bind more strongly to the dimethylated K9 substrate, leading to a decrease in *K*_m_ and an increase in *k*_cat_ with the substrate H3_(1–24)_K4me3-K9me2. Based on these observations we hypothesized that the decrease in *K*_m_ and an increase in *k*_cat_ observed for substrates trimethylated at K4 could also be seen for the full-length enzymes FL-KDM4A and FL-KDM4C since these contain 2 PHD domains known to interact with methylated histone lysines. The enzyme kinetics of FL-KDM4A and FL-KDM4C was determined with H3_(1–24)_K9me3 as a control and compared to H3_(1–24)_K4me3-K9me3 (see figure 7). H3K4me3 in combination with H3K9me3 resulted in an increase in FL-KDM4A demethylation activity, which is significantly higher compared to cc-KDM4A. For FL-KDM4C, a small difference in activity between H3_(1–24)_K9me3 and H3_(1–24)_K4me3-K9me3, comparable to the results for cc-KDM4C was observed.

## Discussion

The major findings of this study are summarized in figure 2, showing the catalytic efficiency constants (*k*_cat_/*K*_m_) for truncated cc-KDM4C and cc-KDM4A along with the full length FL- KDM4C and FL-KDM4A using various synthetic histone tail peptides as substrates. Table 1 and 2 summarize the detailed kinetic analyses of the different histone tail peptide substrates for truncated and full length enzymes, respectively.

**Table 2:** Substrate selectivity of the human FL-KDM4A and FL-KDM4C histone demethylases.

Full Length Enzymes	FL-KDM4A			FL-KDM4C		
Kinetic parameters	*K*_m_	*k*_cat_	*k*_cat_ */K*_m_	*K*_m_	*k*_cat_	*k*_cat_ */K*_m_
**H3_(1–24)_K9me2**	0	0	0	79±10	0.1±0.01	1.3±0.2
**H3_(1–24)_K9me3**	122±31	0.3±0.05	3.1±0.3	57±5	0.7±0.1	12±2
**H3_(1–24)_K4me3K9me3**	45±9	2.2±0.3	50.2±8.3	42±3	1.1±0.2	24±3
**H3_(1–24)_K9me3T11_(ph)_**	0	0	0	0	0	0

The different kinetic parameters *K*_m_, *k*_cat_ and *k*_cat_/*K*_m_ are shown for comparison. 0 means no demethylation could be detected.

Consistent with the results for the truncated versions (cc) of the enzymes, FL-KDM4C exhibited an order of magnitude higher activity towards trimethylated H3K9me3 compared to dimethylated H3K9me2 substrates [21], [22]. The kinetics of substrate H3_(1–24)_K9me2 and H3_(1–24)_K9me3 could not be compared for cc-KDM4A nor FL-KDM4A, since demethylation of H3_(1–24)_K9me2 to H3_(1–24)_K9me1 could not be detected. This is most likely due to the low sensitivity of the FDH-assay in combination with the slow conversion of H3_(1–24)_K9me2 to H3_(1–24)_K9me1 under the conditions applied.

Protein-kinase-C-Related Kinase 1 (PRK1) was previously reported to be responsible for phosphorylation of H3T11, leading to an acceleration of the demethylation reaction rate by KDM4C [6]. This conclusion was based upon Western blot analyses and ChIP assays. In the current *in vitro* study, we see that phosphorylation on threonine 11 prevents the demethylation of H3K9me3 by cc-KDM4A, cc-KMD4C, FL-KDM4C and FL-KDM4A. These observations are in line with the finding that phosphorylation of the adjacent serine 10 H3S10_(ph)_ shields for demethylation of other demethylases [23], but also of cc-KDM4A [24]. Our findings could suggest that the reported increased activity of KDM4C *in vivo* is not occurring on the same histone tail where the phosphorylation of Thr11 is situated, but further studies are needed to reconcile these observations.

Acetylation of K14 H3K9me3-K14_(ac)_ led to different changes in the kinetic parameters of cc-KDM4A and cc-KDM4C with respect to H3K9me3 demethylation. For cc-KDM4A *k*_cat_/*K*_m_ increased 3-fold caused by a decrease in *K*_m_, while for cc-KDM4C it resulted in a 3-fold decrease caused by a decrease in *k*_cat_.

It has been reported that the vast majority of euchromatic genes occupied by H3K9me3 also carry H3K4me3 [7], [19]. On the other hand, H3K9me3 and H3K4me3 have been reported to be mutually exclusive histone modifications associated with the active and repressed chromosomal alleles at most imprinting control regions [25]–[27]. Previously, it has been shown for truncated PHF8 (containing the cc and a PHD domain) that the catalytic activity for H3K9me2 demethylation increases when H3K4 is trimethylated [5]. An X-ray crystallographic study demonstrated that the H3K4Me_3_ moiety interacts with the PHD domain in the enzyme explaining the increased activity through a decrease of *K*_m_ [5].

Our investigation of H3_(1–24)_K4me3-K9me3 showed a 2-fold increase in *k*_cat_/K_m_ for cc-KDM4A and cc-KDM4C compared to H3_(1–24)_K9me3. However, for FL-KDM4A this increase was 17-fold and only 2-fold for FL-KDM4C. This could indicate that trimethylation of K4 is important for demethylation of K9me3 by FL-KDM4A.

**Figure 8:**
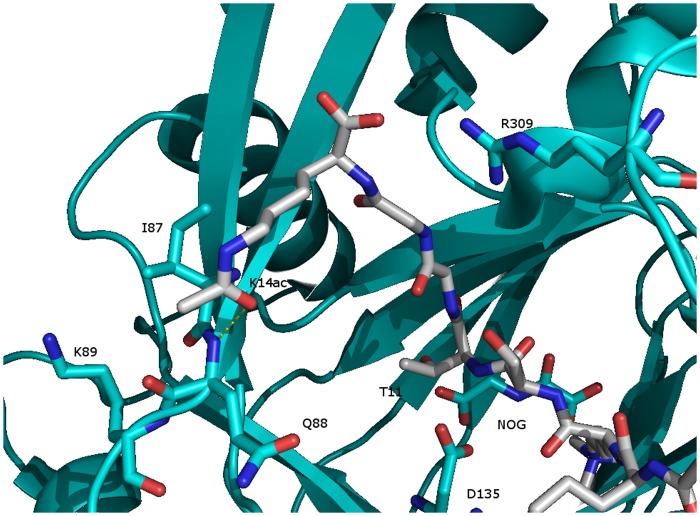
KDM4A co-crystallized with a truncated histone tail H3K9me3K14_(ac)_ (PDB: 2OQ6). It shows that unphosphorylated T11 is deeply embedded in the binding pocket leaving little space for a phosphorylated T11.

To understand the origin of the altered activities, we investigated existing X-ray crystallographic studies in the KDM4 family. The structure (PDB: 2OQ6) of KDM4A [24] co-crystallized with a peptide trimethylated at K9 and acetylated at K14 (Figure 8) shows that T11 is deeply embedded in the binding pocket leaving little space for a phosphorylated T11 in the binding site. Furthermore, electrostatic repulsion from the acidic groups of D135 and NOG (*N*-oxalylglycine, substituting ketoglutarate) would further counteract binding of a H3 histone substrate phosphorylated at T11. Thus, it is not surprising that phosphorylated T11 is shielding for the demethylation of K9me3.

The carbonyl oxygen of the acetyl group on K14 seems to be close enough (3.22 Å) to make a hydrogen bond to the N-H of backbone amide of I87 along with favorable van der Waals interactions with I87. Furthermore, electrostatic repulsion from K89 and/or R309 could disfavor the presence of unacetylated K14. Finally, I87 has van der Waals interactions with L74 and this residue is a methionine (M76) in KDM4C suggesting a reason for the observed different responses between KDM4A and KDM4C towards acetylation of K14.

**Figure 9:**
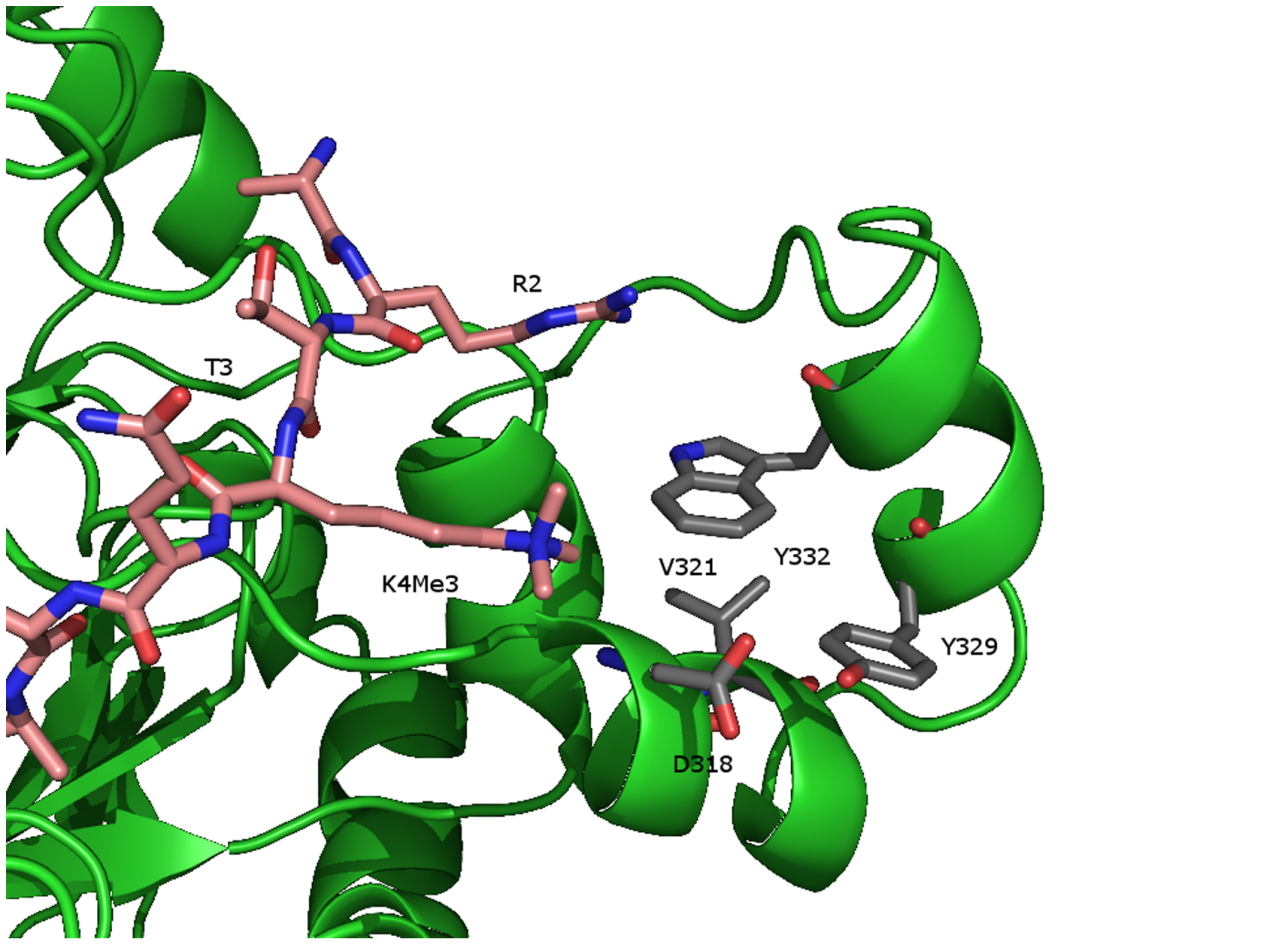
Model of KDM4A created in Schrodinger 2011 Suite. A stable conformation of the trimethylated amino group of K4 in proximity to the “pygo”-box. Here the hydroxyl group of T3 is not buried in the enzyme, leaving space for phosphorylation and still maintain activity as observed.

None of the available crystal structures show how K4 and T3 interact with the catalytic core of the KDM4 enzymes. However, the structure of KDM4A (PDB: 2P5B) co-crystalized with a H3K36 trimethylated peptide shows more of the peptide in a defined conformation. In this structure A31 is in the same distance from K36 that K4 is from K9. Close to residue A31 (figure S6 in File S1) are four residues D318, V321, Y329 and W332 in a box-like arrangement. These are the exact same amino acids in the PHD finger from hPygo1 (D352, V350, Y341and W366) recognizing tri- and dimethylated H3K4 in the hPHD-HD1 complex (figure S7 in File S1). [28] We made a model manually using the protein preparation wizard in Schrodinger 2011 Suite (Schrodinger Inc., Portland, OR), where the rest of the H3 tail was built and energy minimized. As can be seen in figure 9 it is possible to create a stable conformation where the trimethylated amino group of K4 is in proximity to the “pygo”-box; however, the C-terminal domain would have to move in order to accommodate the trimethyl group. In this conformation of the peptide, the hydroxyl group of T3 is not buried in the enzyme, thus there is space for phosphorylation and activity could be maintained as observed.

It is not unlikely that the C-terminal domain is flexible. Structures of KDM4D have shown this part in two very different conformations. Recently, a structure of KDM4D was published [29] resembling the previously published jumonji structures, however in the PDB database two unpublished KDM4D structures (PDB: 3DXU and 3DXT) are deposited showing the C-terminal appendix wrapped over the rest of the demethylase (figure S8 in File S1). While this conformation could be due to crystal packing effects it is tempting to speculate that the C-terminal domain is in fact flexible and can wrap up and make favorable contact with H3K4 and H3R2.

Another possible explanation of the increased activity upon H3K4 trimethylation is through interaction with PHD domains. Previous studies have reported that trimethylated H3K4 can bind the tudor domain of KDM4A, however the functional implication of this interaction is currently unknown [30] and cannot explain the differences in reaction kinetics seen for the catalytic core proteins. However, it cannot be excluded that two peptides bind at the same time and that trimethylated H3K4 on one peptide stimulates the demethylation of H3K9 on another peptide. This mechanism could operate *in vivo* between two neighboring nucleosomes.

Large variations in the enzymatic activities have been reported for cc-KDM4A and cc-KDM4C in the literature depending on constructs and purification procedures. A recent report has shown that by applying a simple affinity purification scheme for Strep (II)-tagged JmjC KDM’s contamination by transition state metal ions is minimized, and KDM4A and KDM4D can be obtained highly pure and with activities substantially higher than previously reported [31]. This is an interesting finding; however, this does not explain our observations. In this study, we have seen variation in catalytic activity up to 25% as a function of different enzyme batches and maximum 2-fold upon different purification procedures.

We have carried out several experiments where Fe^2+^ and other divalent metal ions were removed from the enzymes after purification leading to loss of enzyme activity that was reconstituted upon addition of Fe^2+^. It was found in the most extreme cases that the absolute enzyme activity could be improved by a factor of two, but generally we did not see any significant changes in the enzymatic activity, as a function of metal ion substitutions. These results are to be published in near future (Kristensen et al., unpublished results). Thus, we do not believe that the variations seen here is a result of batch variation or purification methods, also because the various peptides were tested in parallel on the same batch of enzyme. It is tempting to speculate that the variation in enzyme activity reported in the literature is related to how well the C-terminal domain has folded.

Our study indicates that additional PTMs can have an effect not only on the substrate affinity, but also on the catalytic activity in the demethylation of H3K9me3. The increase in *k*_cat_/*K*_m_ upon trimethylation of H3K4 for FL-KDM4A yields the highest enzymatic catalytic efficiency yet reported for this enzyme class. Furthermore, H3_(1–24)_K9me3-T11_(ph)_ had a phosphorylated T11 that shielded for demethylation of K9me3. These results suggest that the catalytic activity for writers and erasers of histone marks can be influenced by the chemical nature of the peptide modifications and this should be investigated in more detail. Definitely, other studies are needed to understand the cross-talk of epigenetic marks. However, we believe that studies like the current one are relevant for understanding the influence of other marks on the same histone tail, and may be highly useful in getting a better understanding of the dynamics of epigenetic marks.

## Materials and Methods

### Enzyme Kinetics

The enzyme kinetics were measured using a Formaldehyde DeHydrogenase Assay (FDH-Assay) and the demethylation of substrates was confirmed when possible by MALDI-TOF-MS, see File S1 for details. The test solution contained 50 mM HEPES (pH 7.5), 50 mM NaCl, 50 µM FeSO_4_, 500 µM α-KG, 500 µM ascorbate, 2 mM NAD, approximately 0.05 unit of FDH and e.g. 4.88 µM KDM4C (enzyme concentration varied between batches). The reference solution was comprised of the same except substrate. The assay was initiated by adding various concentrations of H3 peptide substrates e.g. H3_(1–24)_K9me3 (10–1000 µM) and the increase in NADH fluorescence was measured over 15–30 minutes in 30 second intervals. Data analysis and kinetic values were obtained using programs Excel and GraphPad Prism® 5.0. For details, see File S1.

### Statistics

The standard deviations for *k*_cat_/*K*_m_ were calculated using the statistic law of propagation of errors. Standard errors for Michaelis-Menten Kinetics were automatically given in GraphPad Prism®, with a 95% confidence interval. All enzyme kinetic curves were done in triplicate unless otherwise stated.

## Supporting Information

File S1 (DOCX)

## References

[1] Kouzarides T (2007) Chromatin modifications and their function. Cell 128: 693–705. 17320507 10.1016/j.cell.2007.02.005

[2] Jenuwein T, Allis CD (2001) Translating the histone code. Science 293: 1074–1080. 11498575 10.1126/science.1063127

[3] Cloos PAC, Christensen J, Agger K, Helin K (2008) Erasing the methyl mark: histone demethylases at the center of cellular differentiation and disease. Genes Dev 22: 1115–1140. 18451103 10.1101/gad.1652908 PMC2732404

[4] Li E (2002) Chromatin modification and epigenetic reprogramming in mammalian development. Nature Rev Gen. 3: 662–673. 10.1038/nrg887 12209141

[5] Horton JR, Upadhyay AK, Qi HH, Zhang X, Shi Y, et al (2010) Enzymatic and structural insights for substrate specificity of a family of jumonji histone lysine demethylases. Nat Struct Mol Bio 17: 38–43. 20023638 10.1038/nsmb.1753 PMC2849977

[6] Metzger E, Yin N, Wissmann M, Kunowska N, Fischer K, et al (2008) Phosphorylation of histone H3 at threonine 11 establishes a novel chromatin mark for transcriptional regulation. Nat Cell Bio 10: 53–60. 18066052 10.1038/ncb1668 PMC2878724

[7] Barski A, Cuddapah S, Cui K, Roh T-Y, Schones DE, et al (2007) High-resolution profiling of histone methylations in the human genome. Cell 129: 823–837. 17512414 10.1016/j.cell.2007.05.009

[8] Rust HL, Thompson PR (2011) Kinase Consensus sequences: a breeding ground for crosstalk. ACS Chem. Biol. 6: 881–892. 10.1021/cb200171d PMC3176959 21721511

[9] Couture J-F, Collazo E, Ortiz-Tello PA, Brunzelle JS, Trievel RC (2007) Specificity and mechanism of JMJD2A, a trimethyllysine-specific histone demethylase. Nat Struct Mol Bio 14: 689–695. 17589523 10.1038/nsmb1273

[10] Cloos PAC, Christensen J, Agger K, Maiolica A, Rappsilber J, et al (2006) The putative oncogene GASC1 demethylates tri- and dimethylated lysine 9 on histone H3. Nature 442: 307–311. 16732293 10.1038/nature04837

[11] Peters AHFM, O'Carroll D, Scherthan H, Mechtler K, Sauer S, et al (2001) Loss of the Suv39h histone methyltransferases impairs mammalian heterochromatin and genome stability. Cell 107: 323–337. 11701123 10.1016/s0092-8674(01)00542-6

[12] Narita M, Nuñez S, Heard E, Narita M, Lin AW, et al (2003) Rb-Mediated heterochromatin formation and silencing of E2F target genes during cellular senescence. Cell 113: 703–716. 12809602 10.1016/s0092-8674(03)00401-x

[13] Braig M, Lee S, Loddenkemper C, Rudolph C, Peters AHFM, et al (2005) Oncogene-induced senescence as an initial barrier in lymphoma development. Nature 436: 660–665. 16079837 10.1038/nature03841

[14] Ehrbrecht A, Müller U, Wolter M, Hoischen A, Koch A, et al (2006) Comprehensive genomic analysis of desmoplastic medulloblastomas: identification of novel amplified genes and separate evaluation of the different histological components. J Pathol 208: 554–563. 16400626 10.1002/path.1925

[15] Yang J, Jubb AM, Pike L, Buffa FM, Turley H, et al (2010) The histone demethylase JMJD2B is regulated by estrogen receptor alpha and hypoxia, and is a key mediator of estrogen induced growth. Cancer Res 70: 6456–6466. 20682797 10.1158/0008-5472.CAN-10-0413 PMC4261152

[16] Lohse B, Kristensen JL, Kristensen L, Agger K, Helin K, et al (2011) Inhibitors of Histone Demethylases. Bioorg Med Chem 19: 3625–3636. 21596573 10.1016/j.bmc.2011.01.046

[17] Dai J, Sultan S, Taylor SS, Higgins JMG (2005) The kinase haspin is required for mitotic histone H3 Thr 3 phosphorylation and normal metaphase chromosome alignment. Genes Dev 19: 472–488. 15681610 10.1101/gad.1267105 PMC548948

[18] Govin J, Dorsey J, Gaucher J, Rousseaux S, Khochbin S, et al (2010) Systematic screen reveals new functional dynamics of histones H3 and H4 during gametogenesis. Genes Dev 24: 1772–1786. 20713519 10.1101/gad.1954910 PMC2922505

[19] Bilodeau S, Kagey MH, Frampton GM, Rahl PB, Young RA (2009) SetDB1 contributes to repression of genes encoding developmental regulators and maintenance of ES cell fate. Genes Dev 23: 2484–2489. 19884255 10.1101/gad.1837309 PMC2779743

[20] Wang Y, Kallgren SP, Reddy BD, Kuntz K, López-Maury L, et al.. (2012) Histone H3 Lysine 14 Acetylation Is Required for Activation of a DNA Damage Checkpoint in Fission Yeast. J Biol Chem 287: 6, 4386–4393. 10.1074/jbc.M111.329417 PMC3281674 22184112

[21] Lohse B, Nielsen AL, Kristensen JBL, Helgstrand C, Cloos PAC, et al (2011) Targeting lysine demethylases by truncating the histone 3 tail to obtain selective substrate-based inhibitors. Angew Chem Int Ed 50: 9100–9103. 10.1002/anie.201101849 21919140

[22] Hillringhaus L, Yue WW, Rose NR, Ng SS, Gileadi C, et al (2011) Structural and Evolutionary Basis for the Dual Substrate Selectivity of Human KDM4 Histone Demethylase Family. J Biol Chem 286 (48): 41616–41625. 10.1074/jbc.M111.283689 PMC3308871 21914792

[23] Forneris F, Binda C, Vanoni MA, Battaglioli E, Mattevi A (2005) Human histone demethylase LSD1 reads the histone code. J Biol Chem 280: 41360–41365. 16223729 10.1074/jbc.M509549200

[24] Ng SS, Kavanagh KL, McDonough MA, Butler D, Pilka ES, et al (2007) Crystal structures of histone demethylase JMJD2A reveal basis for substrate specificity. Nature 448: 87–91. 17589501 10.1038/nature05971

[25] Guenther MG, Levine SS, Boyer LA, Jaenisch R, Young RA (2007) A chromatin landmark and transcription initiation at most promoters in human cells. Cell 130: 77–88. 17632057 10.1016/j.cell.2007.05.042 PMC3200295

[26] Yang Y, Li T, Vu TH, Ulaner GA, Hu JF, et al (2003) The histone code regulating expression of the imprinted mouse Igf2r gene. Endocrinology 144: 5658–5670. 12975326 10.1210/en.2003-0798

[27] Lewis A, Green K, Dawson C, Redrup L, Huynh K D, et al (2006) Epigenetic dynamics of the Kcnq1 imprinted domain in the early embryo. Development 133: 4203–4210. 17021040 10.1242/dev.02612

[28] Fiedler M, Sanchez-Barrena MJ, Nekrasov M, Mieszczanek J, Rybin V, et al (2008) Decoding of methylated histone H3 tail by the Pygo-BCL9 Wnt signaling complex. Mol Cell 30: 507–518. 18498752 10.1016/j.molcel.2008.03.011 PMC2726290

[29] Krishnan S, Trievel RC (2013) Structural and Functional Analysis of JMJD2D Reveals Molecular Basis for Site-Specific Demethylation among JMJD2 Demethylases. Structure 21: 98–108. 23219879 10.1016/j.str.2012.10.018

[30] Regha K, Sloane MA, Huang R, Pauler FM, Warczok KE, et al (2007) Active and repressive chromatin are interspersed without spreading in an imprinted gene cluster in the mammalian genome. Mol Cell 27: 353–366. 17679087 10.1016/j.molcel.2007.06.024 PMC2847180

[31] Krishnan S, Collazo E, Ortiz-Tello PA, Trievel RC (2012) Purification and assay protocols for obtaining highly active Jumonji C demethylases. Anal Biochem 420: 48–53.h. 21925481 10.1016/j.ab.2011.08.034

