# Supplementary material for: Posttranslational Modifications of the Histone 3 Tail and Their Impact on the Activity of Histone Lysine Demethylases In Vitro
Source: PLoS One. 2013 Jul 2;8(7):e67653. doi: 10.1371/journal.pone.0067653 (PMC3699631; doi:10.1371/journal.pone.0067653)
Supplement: File S1 — (DOCX) [file pone.0067653.s001.docx]

**Posttranslational modifications of the histone 3 tail and their impact on the activity of histone lysine demethylases *in vitro*.**

*Brian Lohse,^†^* Charlotte Helgstrand,^† (#)^ Jan B. L. Kristensen,^†(#)^ Ulrike Leurs^†^, Paul A.C. Cloos,^§^ Jesper L. Kristensen^†^ and Rasmus P. Clausen^†^*.*

^(#)^These authors contributed equally.

*^†^* Department of Drug Design and Pharmacology, Faculty of Health and Medical Sciences, University of Copenhagen, Universitetsparken 2, 2100 Copenhagen.

*^§^* Biotech Research & Innovation Centre, University of Copenhagen, 2200 Copenhagen, Denmark.

***Corresponding authors:**

E-mail: [bril@sund.ku.dk](mailto:bril@sund.ku.dk), [rac@sund.ku.dk](mailto:rac@sund.ku.dk)

**Supporting Information**

**Table of Contents**

Figure S1: MALDI-TOF-MS for cc-KDM4C with H3_(1-24)_K9me3-T11_(ph)_  S-2

Figure S2: MALDI-TOF-MS for cc-KDM4C with H3_(1-24)_K9me3-K14_(ac)_  S-2

Figure S3: MALDI-TOF-MS for cc-KDM4A with H3_(1-24)_K4me3-K9me3 S-2

Figure S4: MALDI-TOF-MS for cc-KDM4C with H3_(1-24)_K4me3-K9me3 S-2

Figure S5: MALDI-TOF-MS for cc-KDM4A with H3_(1-24)_K4me3 S-3

Figure S6: Molecular modeling of KDM4A with H3K36me3 S-3

Figure S7: Molecular modeling of the hPHD1-HD1 complex S-4

Figure S8: Molecular modeling of two types of KDM4D conformations S-4

General Experimental Details S-5

**
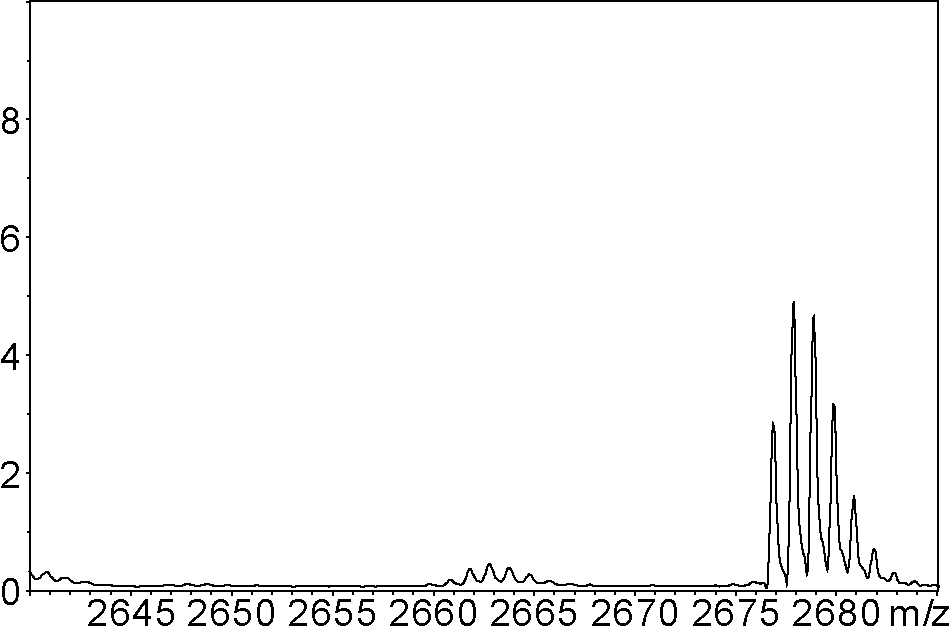

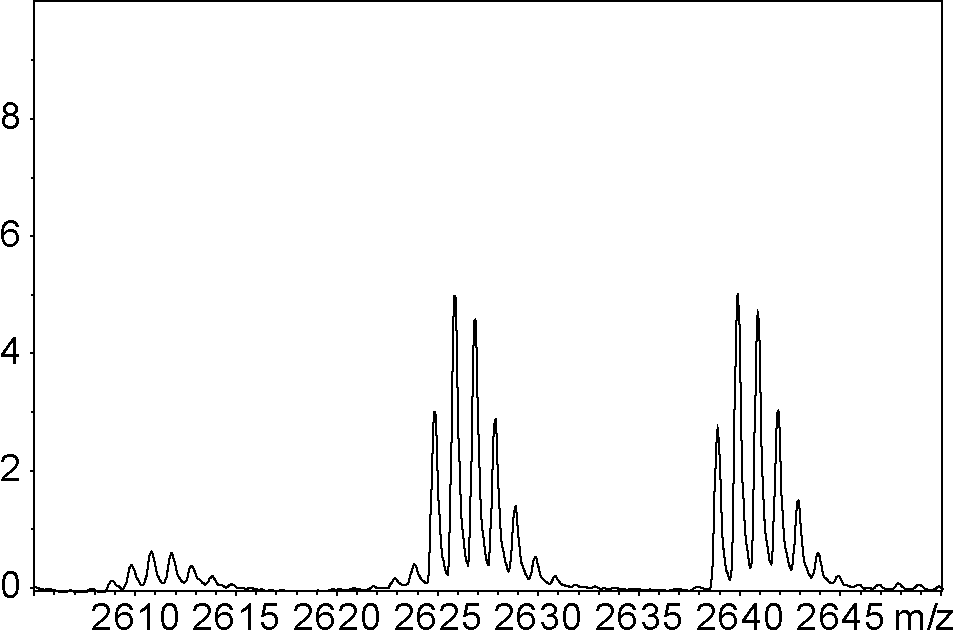
**

**Figure S1 (left) and S2 (right):** MALDI-TOF-MS spectra for cc-KDM4C with substrate H3_(1-24)_K9me3-T11_(ph)_, Mw = 2677; (left figure) and PTM products between mark K9me3 and K14_(ac)_ on cc-KDM4C. MALDI-TOF-MS spectra of H3_(1-24)_K9me3-K14_(ac)_ (Mw = 2639 g/mol) (right figure). The small tops at 2663 m/z in the left figure is an impurity, which could not be removed.

***
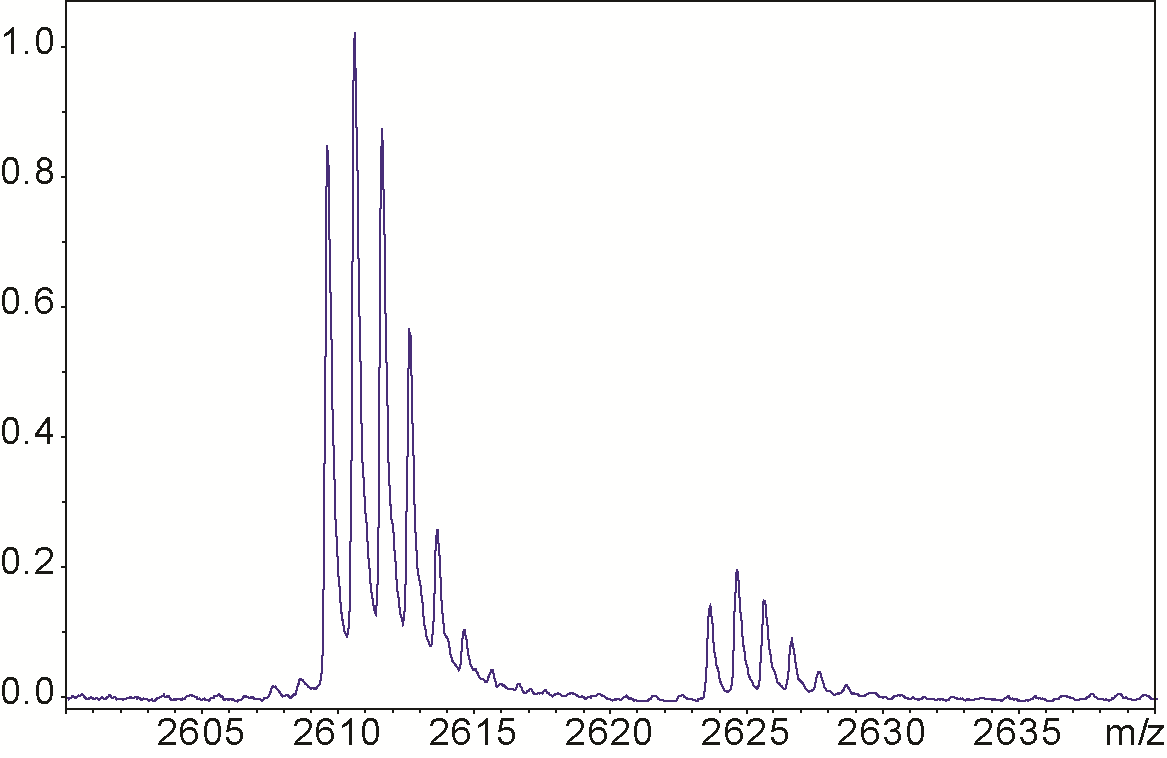
***
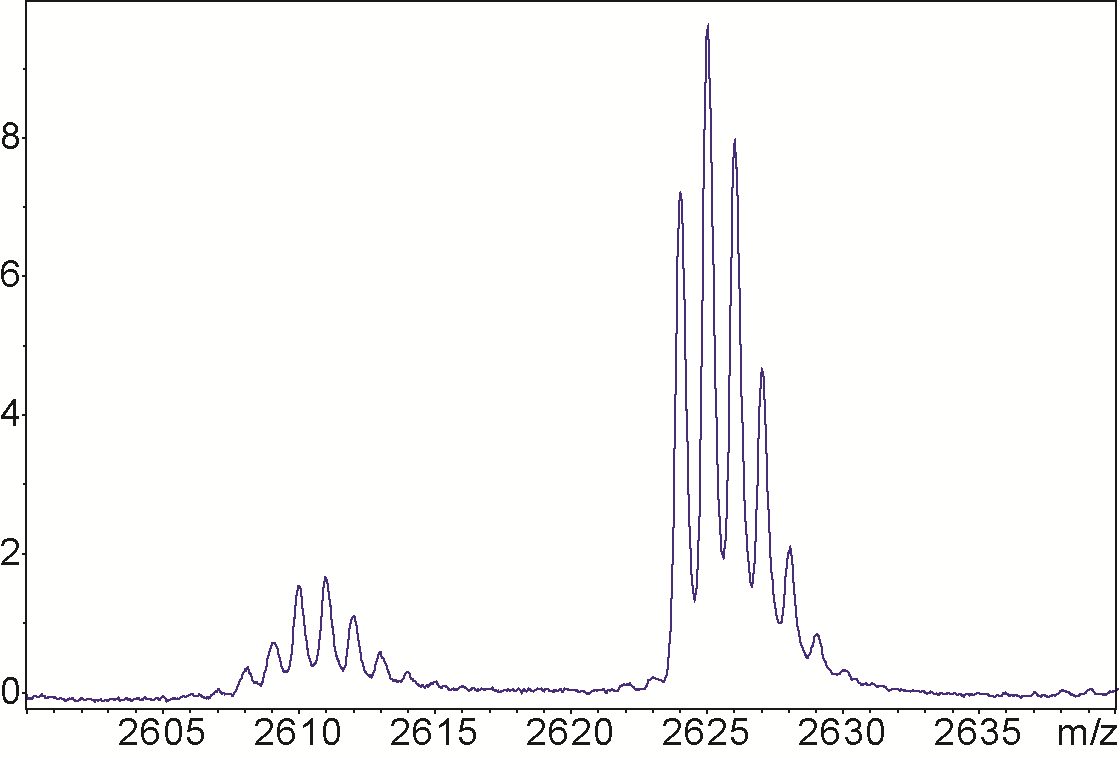


**Figure S3 (left) and S4 (right):** MALDI-TOF-MS spectra of H3_(1-24)_K4me3-K9me3 (Mw = 2638 g/mol) showing PTM product between mark K4me3 and mark K9me3 on cc-KDM4A (S3) and between mark K4me3 and mark K9me3 on cc-KDM4C (S4).

**
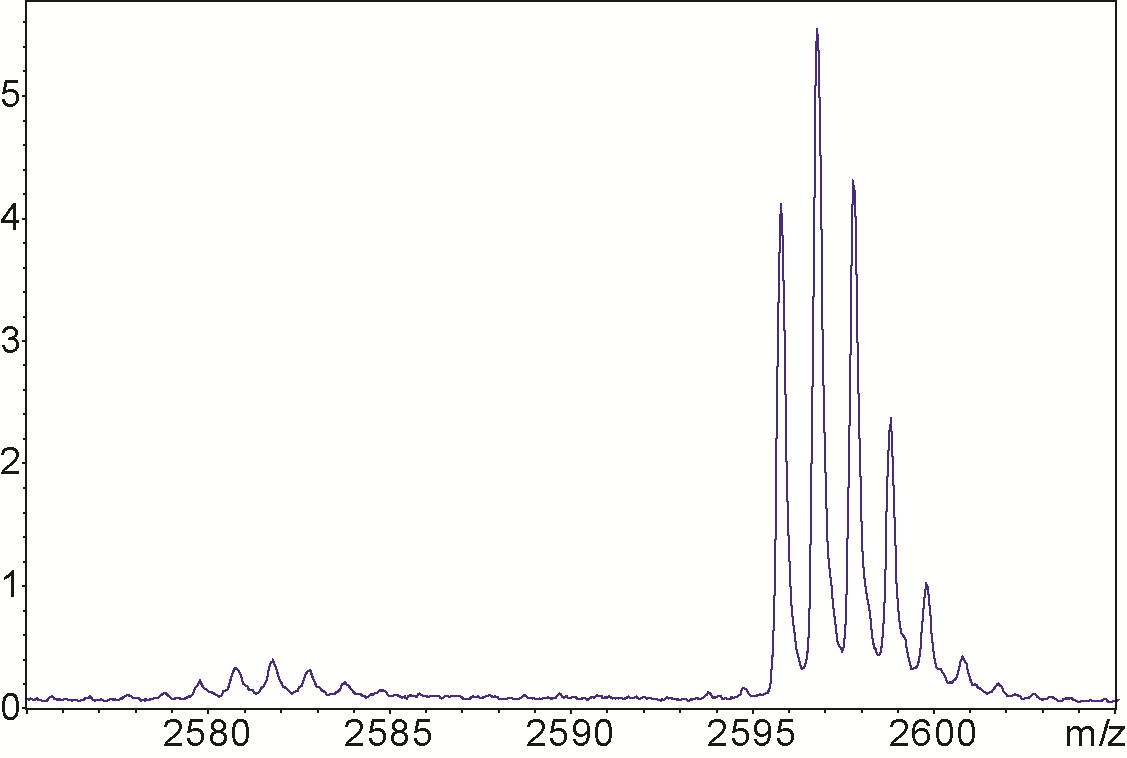
**

**Figure S5:** MALDI-TOF-MS for cc-KDM4A with substrate H3_(1-24)_K4me3, Mw = 2597; The small tops at 2582 m/z in the figure is an impurity, which could not be removed.

**
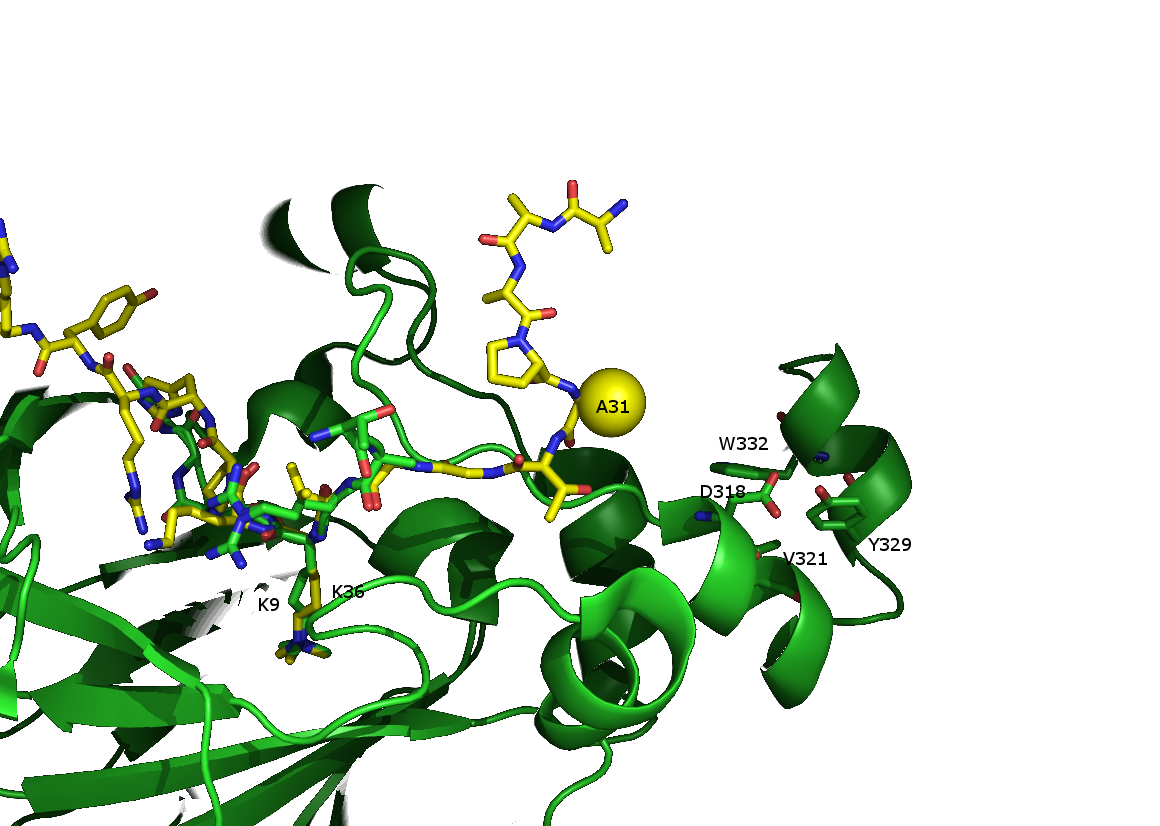
**

**Figure S6:** Box-like arrangement consisting of W332, Y329, D318 and V321 in close proximity to A31 (yellow sphere).


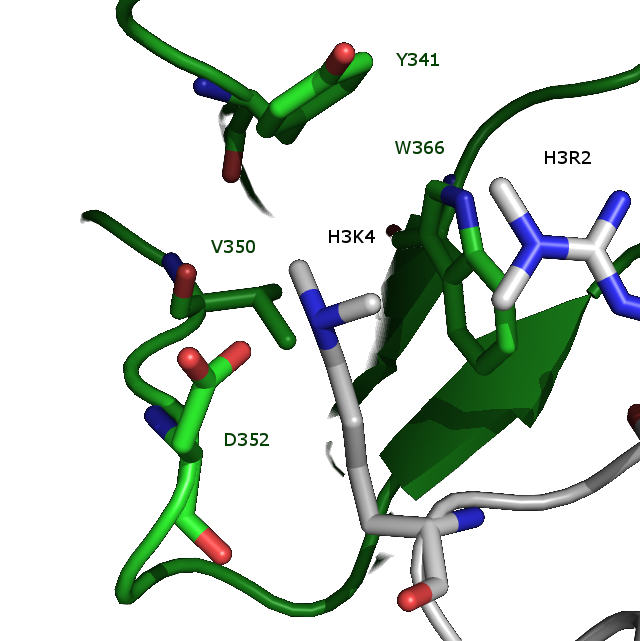


**Figure S7:** Box-like arrangement consisting of W366, Y341, D352 and V350, in close proximity to the two histone tail amino acids H3K4 and H3R2 that are chemically modified through PTMs.

**
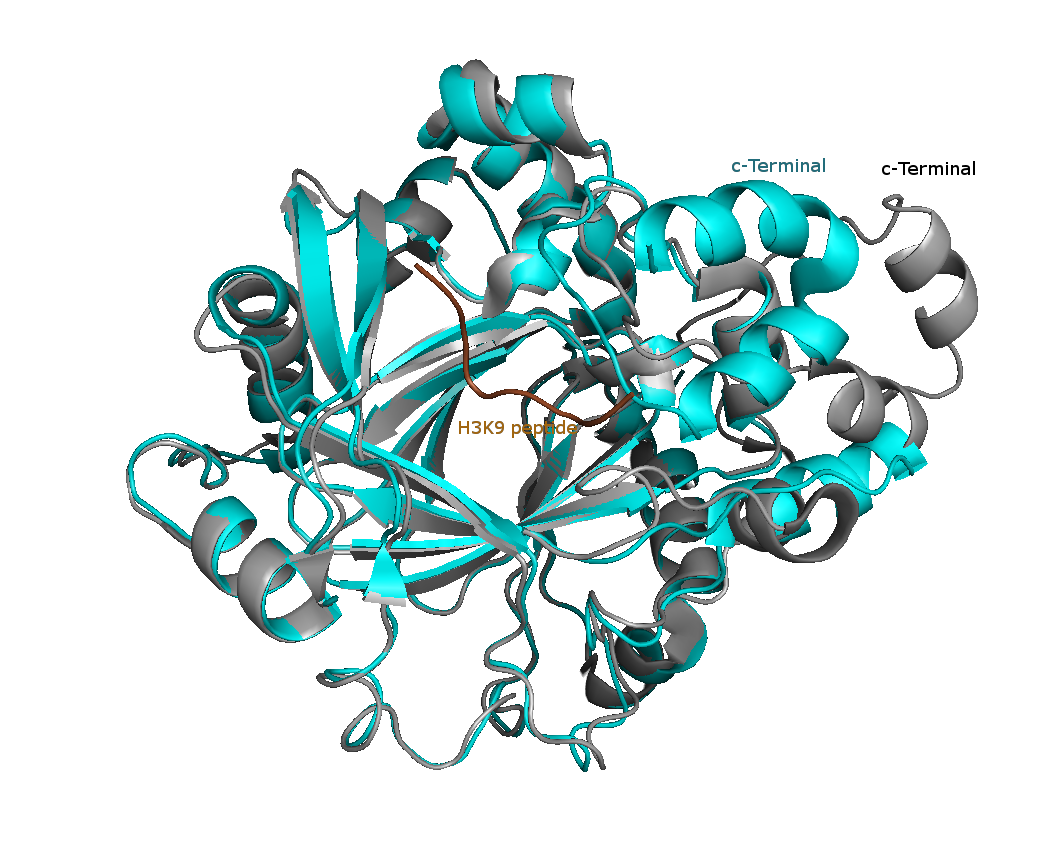
**

**Figure S8:** Two different structures of KDM4D showing that the C-terminal region is flexible and could make contact with H3K4 and H3R2 by twisting and turning the C-terminal (PDB: 3DXU and 3DXT).

**Chemicals and Materials**

All chemicals used for buffers-, co-factors, formaldehyde dehydrogenase from *Pseudomonas putida*, and β–Nicotinamide adenine dinucleotide hydrate from *Saccharomyces cerevisiae* were obtained from Sigma-Aldrich. Standard amino acid building blocks were purchased from either Genscript or Iris Biotech Gmbh, methylated Fmoc-Lys-OH was from Novabiochem. Solvents for MALDI-TOF were of HPLC-grade and from Merck. EDTA free protease inhibitor cocktail tablets used in protein purification were from Roche. The plasmid coding for cc-KDM4A and cc-KDM4C was generously donated by the Biotech Research and Innovation Centre (BRIC). All reagents and solvents for the synthesis were purchased from Sigma-Aldrich and used without further purification.

**Synthesis of Peptides**

Synthesis of peptides and Fmoc building blocks was performed as described in Lohse *et al*.^21^ All Peptides were more than 95% pure according to LC-MS.

**Enzyme Kinetics**

Test for activity of enzyme using the fluorescence FDH assay, determination of *V*_max_ and *K*_m_ of enzymes with substrate, expression and purification of cc-KDM4A and cc-KDM4C software and parameters for the FDH coupled assay, see Lohse *et al*.^21^

**Protein expression and purification of Full Length FL-KDM4A and FL-KDM4C**

Recombinant *baculoviridae* encoding the full Open Reading Frame (ORF) of FL-KDM4A and FL-KDM4C were generously donated from the Biotech Research and Innovation Centre (BRIC), University of Copenhagen, Denmark. Recombinant protein was expressed by adding High Titer Stocks to High Five (Trichopulsia ni) insect cells in suspension culture. The cell cultures were harvested by centrifugation after ~ 60 hours and the cells were resuspended in hypotonic buffer (25 mM Hepes-KOH, pH 7.7, 1.5 mM MgCl, 5 mM KCl) supplemented with one protease inhibitor cocktail tablet per 500 ml expression (Roche, EDTA-free). The resuspended cells were frozen at -20^o^C.

The expressed proteins both had an N-terminal His_6_-tag and in addition KDM4C also had an N-terminal FLAG-tag. Both proteins were purified by IMAC using Talon FF (Clontech) cobalt resin according to the following protocol; resuspended cells were thawed on ice, 100 ml hypotonic buffer was added per 500 ml expression volume and the cells were incubated with stirring for 30 min. at 4˚C, followed by a brief (30 s per 500 ml culture volume) sonication. β-mercaptoethanol (BME, 2 mM final concentration), Triton X-100 (0.2 % final concentration) and NaCl (300 mM final concentration) were added and the cell suspension was stirred for another 30 min at 4˚C. The lysate was cleared by centrifugation at 50.000 g for 60 minutes. The cleared lysates were adjusted to 5 mM imidazole and incubated with Talon resin (1 ml /500 ml expression) for 60 minutes. The resin was allowed to sediment, the lysate removed and the resin was transferred to PD-10 gravity flow columns (GE Healthcare). The resin was washed with 10 column volumes of binding buffer (50 mM Hepes-KOH, pH 7.7, 300 mM NaCl, 5 mM imidazole, 10% glycerol, 0.05 % Triton X-100, 2 mM BME) and eluted with 2 x 2.5 ml elution buffer (50 mM Hepes-KOH, pH 7.7, 300 mM NaCl, 100 mM imidazole, 10 % glycerol, 0.05 % Triton X-100, 2 mM BME). Each eluted fraction has its buffer changed to 50 mM Hepes pH 7.5, 300 mM NaCl, 10 % glycerol using a PD-10 desalting column. The protein was concentrated to 6-7 mg/ml (FL-KDM4A) or 10-12 mg/ml (FL-KDM4C). Despite the buffer change it is likely that small amounts of Triton X-100 remained in the protein samples. Triton X-100 has a significant absorbance at 280 nm, so the protein concentration was determined by Bradford assay using a BSA standard curve. The protein purity was approximately 70 % as judged from SDS-PAGE. The concentrated protein was aliquoted and stored at -80^o^C until assays were performed.


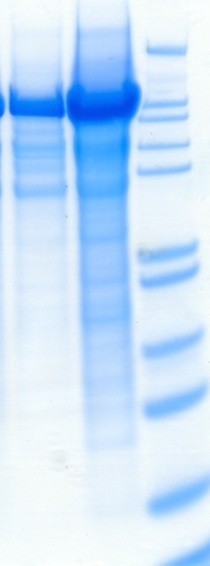


1 2 3

FL-KDM4A purity, Lane 1: an eluted fraction after buffer exchange, lane 2: concentrated sample 7 mg/ml, lane 3, Mark 12 MW marker (Invitrogen^TM^). The impurities are most likely of cellular origin.


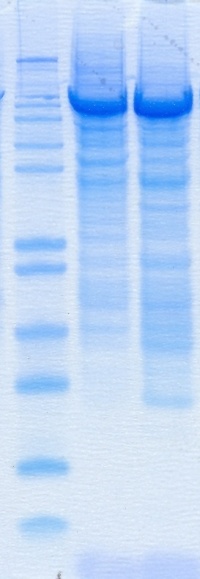


1 2 3

FL-KDM4C purity. Lane 1, Mark 12 molecular weight marker (Invitrogen^TM^), lanes 2 and 3, 2 eluted fractions from the Talon affinity purification. The impurities are most likely of cellular origin.

**MALDI-TOF-MS (truncated enzymes)**

The reaction mixture was diluted 1: 9 with a saturated solution of α-cyano-4-hydroxycinnamic acid (Aldrich) in a mixture of 65 % acetonitrile and 35 % MilliQ water containing 0.1 % TFA. 1 µl of the sample was spotted onto the target and air dried. The MALDI-TOF MS was carried out on an Ultraflex TOF/TOF (Bruker) operated in positive ion mode with an Ion source voltage of 25 kV, a lens voltage of 7.5 kV and a reflector voltage of 26.3 kV. The system was run in deflection mode with a mass suppression of 500 Da. The data analysis was carried out using the FlexAnalysis software (Bruker), baseline subtraction and smoothing of the curves was applied.

**MALDI-TOF-MS efforts on Full Length enzymes**

**General procedures for recording MALDI-TOF spectra**

Matrix solution was prepared by making a saturated solution of α-cyano-4-hydroxycinnamic acid (Aldrich) in a mixture of 65 % acetonitrile and 35 % MilliQ water containing 0.1 % TFA. 1 µl spotting volumes were used for all samples.

The spectra were recorded on an Ultraflex TOF/TOF (Bruker) operated in positive ion mode with an Ion source voltage of 25 kV, a lens voltage of 7.5 kV and a reflector voltage of 26.3 kV. The system was run in deflection mode with a mass suppression of 500 Da. The data analysis was carried out using the FlexAnalysis software (Bruker), smoothing of the curves was applied.

**Processing of Full-length enzyme assay mixtures for MALDI analyses**

Samples were first attempted spotted directly using a 1: 9 ratio. Secondly the sample was diluted 1: 9 with 0.1 % TFA and further diluted 1: 9 with matrix and spotted. None of these procedures yielded any detectable product- or substrate peaks in the spectra. Next it was attempted to desalt the samples directly. 10 µl of the reaction mixtures was desalted using µC-18 Zip Tips (Millipore), following the manufacturer’s instructions, the sample was eluted in matrix and spotted directly. Neither the crude sample, nor sample diluted 1: 9 with 0.1 % TFA, gave any detectable product- or substrate peaks in the MS-spectra. Peptides solubilized in reaction buffer were subjected to the same treatment and were used as controls; all peptides were detected with the expected masses.

Secondly it was attempted to fully denature the protein in order to remove any interfering effects from this on the substrate/products. The sample was mixed in a 1: 1 ratio with 4 M urea and incubated for 1 h, either at RT. or at 70°C. The sample was subsequently desalted and processed as described above. No product- or substrate peaks were observed in the resulting spectra.

**Statistics**

The standard deviations for *k*_cat_/ *K*_m_ were calculated using the statistic law of propagation of errors. Standard errors for Michaelis-Menten Kinetics were automatically given in GraphPad Prism®, with a 95 % confidence interval. All enzyme kinetic curves were done in triplicate unless otherwise stated.
